# The effects of patient cost sharing on inpatient utilization, cost, and outcome

**DOI:** 10.1371/journal.pone.0187096

**Published:** 2017-10-26

**Authors:** Yuan Xu, Ning Li, Mingshan Lu, Elijah Dixon, Robert P. Myers, Rachel J. Jelley, Hude Quan

**Affiliations:** 1 Beijing YouAn Hospital, Capital Medical University, Beijing, China; 2 Department of Community Health Sciences, University of Calgary, Calgary, Alberta, Canada; 3 Department of Economics, University of Calgary, Calgary, Alberta, Canada; 4 Division of General Surgery, Department of Medicine, University of Calgary, Calgary, Alberta, Canada; 5 Liver Unit, Division of Gastroenterology and Hepatology, Department of Medicine, University of Calgary, Calgary, Alberta, Canada; Old Dominion University, UNITED STATES

## Abstract

**Background:**

Health insurance and provider payment reforms all over the world beg a key empirical question: what are the potential impacts of patient cost-sharing on health care utilization, cost and outcomes? The unique health insurance system and rich electronic medical record (EMR) data in China provides us a unique opportunity to study this topic.

**Methods:**

Four years (2010 to 2014) of EMR data from one medical center in China were utilized, including 10,858 adult patients with liver diseases. We measured patient cost-sharing using actual reimbursement ratio (RR) which is allowed us to better capture financial incentive than using type of health insurance. A rigorous risk adjustment method was employed with both comorbidities and disease severity measures acting as risk adjustors. Associations between RR and health use, costs and outcome were analyzed by multivariate analyses.

**Results:**

After risk adjustment, patients with more generous health insurance coverage (higher RR) were found to have longer hospital stay, higher total cost, higher medication cost, and higher ratio of medication to total cost, as well as higher number and likelihood that specific procedures were performed.

**Conclusion:**

Our study implied that patient cost-sharing affects health care services use and cost. This reflects how patients and physicians respond to financial incentives in the current healthcare system in China, and the responses could be a joint effect of both demand and supply side moral hazard. In order to contain cost and improve efficiency in the system, reforming provide payment and insurance scheme is urgently needed.

## Introduction

With health care expenditures growing at an increasing speed worldwide, many health care systems are looking into health insurance reforms to contain costs [1–3]. One main cost containment strategy is to rely on patient cost-sharing (patients pay a portion of health care costs not covered by health insurance). It has been argued that the demand of health services could vary with the level of patient cost-sharing: patients with higher health insurance coverage tend to overuse health services, and patients with lower insurance coverage may underuse necessary and otherwise unaffordable care–i.e., the moral hazard effect [4, 5]. On the supply side, health insurance coverage may also influence physicians’ decision-making on health services use: physicians may tend to over-treat patients with more generous health insurance coverage, and under-treat those with poor health insurance coverage–i.e., the supply induced demand effect [1, 6].

In practice, treatment decision is likely made by patient and health care provider jointly. The final treatment decision, therefore, is subject to both demand side moral hazard and supplier-induced demand effects. However, it is difficult to segregate one from the other. Taking China’s health system as an example, patient cost-sharing reflects financial incentives not only for patients but also for health providers (hospitals and physicians), as hospitals are allowed to retain any surplus gained and use this to pay for instance, staff bonuses, which is a large proportion of their incomes [7]. Therefore, the overall effect of patient cost-sharing on health use and cost would reflect a joint effect of health providers’ and patients’ reactions to financial incentives.

For the purpose of evidence-based policy making in health care insurance reform, a key empirical question is: what are the impacts of patient cost-sharing on health care utilization, cost, and patient outcome? In spite of the large number of health economics studies assessing this question [8–12], most of these studies have limitations. Being the only randomized control trial on this topic, the RAND health insurance experiment (HIE) is a classic investigation that provided solid evidence on price elasticity of health care demand [13]. However, the HIE participants were assigned to different insurance plans which failed to count the individual patient’s actual cost-sharing [13, 14]. Many other studies in the literature have had to use data from non-experimental settings, suffering from selection bias. For example, in health insurance markets such as the U.S. there are variations in terms of health insurance coverage (patient cost-sharing), as patients may choose to enroll in different health insurance plans based on their health and expected demand of health services. While in other health insurance markets such as Canada, under universal health insurance coverage there is no variation in cost-sharing for services included in the public health insurance. Finally, it is a common limitation in most of these studies that detailed information on patient cormobidities and disease severity is not available to have proper risk adjustment.

The Chinese healthcare system and its electronic medical record (EMR) data provide us a unique opportunity to contribute to the existing literature and shed light on the above question. Through several large-scale health care reforms in the past decades, China has established a universal health care system in which there are large variations in actual coverage/reimbursement not only among but also within various health insurances [7, 15, 16]. Unlike the private health insurance market in which people choose to enroll in different private insurance plans, most Chinese are covered by different public health insurance plans based on their eligibility (which is determined by occupation, residence, etc.). In other words, there are large cost-sharing variations that can be studied with limited selection bias in the Chinese system. Furthermore, the EMR in China has been rapidly growing since 2006, and now most hospitals in China are using EMRs. Chinese EMRs contain not only rich clinical (including diagnostic and treatment procedure) information but also detailed and itemized billing data.

In this study, we examined the impacts of patient cost-sharing on hospitalization cost, medication cost, treatment/procedures, and in-hospital mortality. Unlike most existing studies using data that measures patient cost-sharing by type of health insurance, we used actual reimbursement ratio (RR, defined as percentage of reimbursed cost in total treatment cost), which was derived using detailed financial information in our EMR data. Given the large variations in RR both among and within different health insurances in China, using RR estimate has allowed us to better capture financial incentive than if using type of health insurance. In addition, the rich clinical information in our EMR data has also allowed us to employ a risk adjustment methodology with a wide range of risk factors to adjust for disease severity and comorbidities.

## Method

### Study setting

#### Liver diseases in China

Liver disease, including viral hepatitis (e.g., Hepatitis B virus, HBV), cirrhosis and primary liver cancer (PLC), is regarded as a large contributor to burden of disease worldwide. Such disease burden is especially high for the Chinese population [17]. In China, about 97 million people are HBV carriers [18]; and at least 20 million patients have chronic HBV infection with or without cirrhosis and/or PLC [17, 18]. This has resulted in high volumes of hospitalized patients with liver disease. Between 2006 and 2010, about 1.2% of inpatients were admitted due to cirrhosis (mainly hepatitis cirrhosis) in hospitals in Beijing [19].

#### EMR development in China

Researchers worldwide have recognized the potential value of EMR and tremendous efforts are underway to advance EMR research [20–22]. EMRs are greatly advantageous in that they provide large geographical coverage and are easily accessible, as well as providing rich and timely longitudinal clinical data for research purposes [23, 24]. In China, developing EMRs has been identified as a primary focus in on-going health care reforms, and the implementation of EMRs has been rapidly growing since 2006 [23]. The coverage, functionality, and interoperability of EMRs will continue to be greatly improved as these developments take place. Currently, most hospitals in China use EMRs; and these EMR systems continue to collect massive amounts of individual clinical and billing information [23, 25].

#### Healthcare system in China

Through recent health reforms, China has established nearly universal health insurance coverage for its population. Under the current public health insurance system, there are three main components: Urban Employee Basic Medical Insurance (UEBMI) which was launched in 1998, New Cooperative Medical Scheme (NCMS) launched in 2003, and Urban Resident Basic Medical Insurance (URBMI) launched in 2007 [26]. Although the health insurance is predominantly public, patients do not have options to choose which public health insurance she/he enrolls. A citizen’s public health insurance type is determined by residency (urban or rural) and employment status (employed or unemployed). However, the insurance policies and coverage, including deductibles, limits, and reimbursement rates for different drugs and services, vary widely not only within but also among the insurance schemas by age, length of employment, and working sectors. Therefore, RR may not be an exogenous variable, but we believe the selection effect of the variation of RR is less in China compared with that in western countries with different arrangements in both insurance and labor markets. Our study adopts similar methodology as that in several previous studies to address the impacts of health insurance (measured by RR) on health care services utilization and cost in China [27–29].

Although China’s public hospitals deliver more than 90% of the country’s health service output [26], since the late 1980s, the government has made large cut backs to its financial input to public hospitals. After the large financial input cut, China’s public hospitals were permitted to retain surplus from drug or services sales [30, 31]. In China prescription drugs are predominantly distributed and sold in hospitals, which accounts for over 70% of all drugs sold and distributed [32], as well, the hospitals are allowed to mark-up drug price (usually 15% margin on drugs) [28] to gain profit for operating costs and retaining surplus. Hospitals pay bonuses to their chief executive officers (CEOs) as well as physicians using the retained surplus. In China, public hospital CEOs’ and physicians’ income consists of a salary and bonuses. Their salary levels are set very low with bonuses accounting for the majority of their income.

Under the current system, there are strong financial incentives for hospitals to become profit-driven to maximize their incomes. Methods to over-value service costs include increasing medication prescriptions or medical material use, as physician workload-related services such as visits and work hours have relatively low-value in terms of cost [26, 30, 31]. For example, transcatheter arterial chemoembolization (TACE) is a common micro-invasive treatment for patients with liver cancer or cirrhosis and can act as an alternative treatment for patients who are not suitable or refused for liver surgery [33]. However, TACE is not a curable method for liver cancer or cirrhosis and is often repeatedly performed upon disease reoccurrence [34]. The catheter material cost is the main contributing factor to the overall cost of TACE. Because pricing policies including over-valuing material costs allow physicians and hospitals to retain a certain margin of the catheter cost, and this impacts physician income. Thus, there are financial incentives for physicians to suggest patients with liver cancer or cirrhosis to choose TACE as a first treatment and to undergo more TACE procedures when the disease reoccurs.

### Data and analysis method

#### Data and sample selection

This study was approved by the YouAn Hospital Research Board of Ethics and the Health Research Ethics Board at University of Calgary (Ethic committee’s reference number: REB14-0815). The EMR data used in this study was derived from Beijing YouAn hospital, which is one of the leading teaching hospitals specializing in liver diseases in China, treating over 300,000 patients from all-over China annually. In 2008, the EMR system was officially implemented in YouAn hospital. EMR data are inputted by physicians and regularly audited by the medical records department in the hospital, as its quality is a part of the physicians’ monthly performance evaluations.

The EMR financial data is directly drawn from the hospital billing system and reflected in the patient’s actual total and out-of-pocket expenses of hospitalization, which includes itemized costs that occur in hospital, such as fees for bed occupation, medication, and surgery or procedures.

For our study sample, we included in-patients with common liver diseases including cirrhosis, PLC, viral hepatitis, alcoholic hepatitis or fatty liver, who were admitted to Beijing YouAn hospital between January 1st, 2010 and September 30th, 2014, 18 years old or older, and consented to use their EMRs for research. We had no access to any patient identifying information as part of the study. In our previous study, we developed and validated a data extraction method for a Chinese EMR, and we defined 40 liver disease severity and comorbidity variables using this extraction method [35].

Using the financial information in our EMRs, we constructed the actual reimbursement ratio (RR) and used this as a measure of actual patient cost-sharing [27]. RR was defined as the ratio of the risk-pooling fund paid costs to total hospital costs: RR = (TC—OOP)/TC * 100%, where TC denotes total hospital cost, and OOP denotes out-of-pocket cost. When RR = 0%, this indicates that the total hospital cost is completely self-paid, and when RR = 100%, this indicates that the total hospital cost is fully reimbursed.

Reimbursement ratio in our data ranged between 0 and 1. Because public health insurance is not portable in China, patients from other provinces have to first pay the hospital costs completely out-of-pocket (RR = 0 in our billing data). To avoid mixing patients who did not have any insurance with those who required reimbursement in their resident provinces/cities (RR is 0 for both cases), we excluded patients with RR = 0 from our sample. This resulted in 10,858 adult patients with liver disease in our study sample.

#### Analysis method

We estimated the impacts of patient cost-sharing using the following model specifications:

$$Y_{i}=\alpha +\beta RR_{i}+\tau Procedure_{i}+\delta L_{i}+\gamma X_{i}+\varepsilon$$ (1)

where Y_*i*_ indicates cost, health care utilization, and patient outcome. Cost measures included total hospital cost (TC, log transformed), medication cost (MC, log transformed), and ratio of medication cost to total cost (RMT, log transformed). As discussed earlier, our hypothesis is that patients with a higher RR have higher hospital costs, i.e. higher TC, MC and RMT. All cost values were inflated to 2010 RMB.

Health care utilization was measured using hospital length of stay (LOS, log transformed), TACE use (TACE = 1 if TACE was used, TACE = 0 if TACE not used), and number of TACE procedures (nTACE, 1 for having one TACE, 2 for having two times TACE, 3 for having 3 times TACE, and so forth). As discussed earlier, our hypothesis is that patients with higher RR will have both a higher likelihood of undergoing TACE and increased number of TACE procedures, as well as a longer hospital LOS. Finally, outcome was measured using in-hospital mortality (1 for yes, 0 for no).

The control variables in our regression models included the following: RR_*i*_, which was the reimbursement ratio of patient *i* and key variable of interest of our analyses; Procedure_*i*_, which indicated whether the patient *i* underwent a major procedure (i.e., hepatectomy or TACE); L_*i*_, which referred to the common types of liver disease including hepatitis B and C, alcoholic hepatitis, fatty liver, cirrhosis, and PLC (all were included as separate dummy variables); as well as X_*i*_, a vector of patient-level risk adjustors.

Based on our earlier study comparing the performance of various risk adjustment models for liver disease using the same data set, the model combining the liver disease severity score (i.e., the model for end-stage liver disease and sodium (MELDNa)) and comorbidity index (i.e., Elixhauser comorbidity index) as risk adjustors showed the best prediction performance [36]. Therefore, we used the following patient-level risk adjustors (X_*i*_) in our model: patient-level demographic information (age, sex, and admission status), the MELDNa score and Elixhauser comorbidity index.

We assessed the effect of RR on the hospital LOS, TC, MC, and RMT through applying a generalized linear regression model. We estimated the effect of RR on the likelihood of using TACE among cirrhosis and PLC patients using a logistic model, and the number of TACE procedures among patients who underwent at least one TACE during the study period using a negative binomial regression model. The effect of RR on the hospital mortality was analyzed using a logistic regression model (Table 1).

**Table 1:** Outcomes, the corresponding explanatory variables and the regression models.

Dependent Variable	Independent Variable	Regression
LOS	RR, age, sex, admission status, Elixhauser comorbidities, MELDNa, major procedure/surgery (hepatectomy and TACE), liver disease categories (PLC, cirrhosis, viral hepatitis, alcoholic hepatitis, and fatty liver)	Generalized linear
TACE (yes/no)	RR, age, sex, admission status, Elixhauser comorbidities, MELDNa, major procedure/surgery (hepatectomy and TACE), liver disease categories (viral hepatitis, alcoholic hepatitis, and fatty liver)	Logistic
nTACE	RR, age, sex, admission status, Elixhauser comorbidities, MELDNa, major procedure/surgery (hepatectomy and TACE), liver disease categories (viral hepatitis, alcoholic hepatitis, and fatty liver)	Generalized linear
TC	RR, age, sex, admission status, LOS, Elixhauser comorbidities, MELDNa, major procedure/surgery (hepatectomy and TACE), liver disease categories (PLC, cirrhosis, viral hepatitis, alcoholic hepatitis, and fatty liver)	Generalized linear
MC	RR, age, sex, admission status, LOS, Elixhauser comorbidities, MELDNa, major procedure/surgery (hepatectomy and TACE), liver disease categories (PLC, cirrhosis, viral hepatitis, alcoholic hepatitis, and fatty liver)	Generalized linear
RMT	RR, age, sex, admission status, LOS, Elixhauser comorbidities, MELDNa, major procedure/surgery (hepatectomy and TACE), liver disease categories (PLC, cirrhosis, viral hepatitis, alcoholic hepatitis, and fatty liver)	Generalized linear
Mortality	RR, age, sex, admission status, Elixhauser comorbidities, MELDNa, major procedure/surgery (hepatectomy and TACE), liver disease categories (PLC, cirrhosis, viral hepatitis, alcoholic hepatitis, and fatty liver)	Logistic

LOS: length of stay at hospital; RR: reimbursement ratio; MELDNa: model for end-stage liver disease and sodium; TACE: transcatheter arterial chemoembolization; PLC: primary liver cancer; nTACE: number of TACE; TC: total cost; MC: medication cost; RMT: ratio of medication cost to total cost; RMT: ratio of medication to total hospital cost.

## Results

As shown in Table 2, the mean age of our study sample was 46 years old, and 49.0% (5,313) of the study population was male. Urgently admitted patients account for 13.9% (1,508). 4.9% (527) patients underwent hepatectomy or TACE. RR ranged from 0.1 to 1.0, and the average RR of the patients was 0.68 (SD 0.19). Among the 10,858 liver disease patients, 20.4% (2,219) had PLC, 34.4% (3,734) had cirrhosis, and 83.8% (9,094) had hepatitis. Among the 4,736 cirrhosis and/or PLC patients, 434 underwent TACE. The five most common comorbidities were: diabetes (with or without complications) 30.5% (3,212), hypertension (complicated or uncomplicated) 27.8% (3,015), anemia (blood loss or deficient) 26.4% (2,861), solid tumors 21.7% (2,357), and alcohol abuse 17.7% (1,926). The mean MELDNa score of the liver disease patients was 11.5 (SD 4.9). The overall in-hospital mortality was 5.5% (600), the mean LOS was 20 days (SD 21 days), the average total cost was 28,908.3 (SD 44,249.2) RMB, the mean medication cost was 17,035.1 (SD 27,259.0) RMB and the mean RMT was 0.49 (SD 0.19).

**Table 2:** Main characteristics of patients with liver disease.

Variable	Definition	Mean (SE)
RR	Numerical variable, RR = reimbursement divide by total hospital cost, therefore it was between 0 and 1.	0.68 (0.19)
Age	Numerical variable, the unit was year. The minimum age was 18 years.	45.9 (15.5)
MELDNa score	Numerical variable ranged from 6.43 to 40; MELDNa = MELD + (140—Na)(1–0.025*MELD); MELD = 3.8 ln(total bilirubin) + 11.2 ln(INR) + 9.6 ln(creatinine) + 6.43; the units for serum level of total bilirubin, creatinine, and INR are mg/dL, mg/dL and 1, respectively.	11.5 (4.9)
LOS	Numerical variable, it was calculated by subtracting discharge date by admission date, the unit was day.	19.94 (21.0)
Total cost	Numerical variable, the unit was Yuan, RMB.	28908.3 (44249.2)
RMT	Numerical variable, RMT was calculated by dividing total hospital cost by medication cost and was between 0 and 1.	0.49 (0.19)
Medication cost	Numerical variable, the unit was Yuan, RMB	17035.1 (27259)
Death	Dummy variable = 1 if patient was dead in hospital, 0 otherwise	0.055 (0.002)
Sex	Dummy variable = 1 if patient was male, 0 female	0.489 (0.005)
Admission status	Dummy variable = 1 if patient was urgently admitted, 0 otherwise	0.139 (0.003)
Hepatectomy	Dummy variable = 1 if patient underwent hepatectomy in hospital, 0 otherwise	0.008 (0.001)
TACE	Dummy variable = 1 if patient underwent TACE in hospital, 0 otherwise	0.040 (0.002)
Primary liver cancer	Dummy variable = 1 if patient was diagnosed as primary liver cancer, 0 otherwise	0.204 (0.004)
Cirrhosis	Dummy variable = 1 if patient was diagnosed as cirrhosis, 0 otherwise	0.344 (0.005)
Hepatitis B	Dummy variable = 1 if patient was diagnosed as hepatitis B, 0 otherwise	0.583 (0.005)
Hepatitis C	Dummy variable = 1 if patient was diagnosed as hepatitis C, 0 otherwise	0.032 (0.002)
Alcoholic hepatitis	Dummy variable = 1 if patient was diagnosed as alcoholic hepatitis, 0 otherwise	0.079 (0.003)
Congestive heart failure	Dummy variable = 1 if patient was diagnosed as congestive heart failure, 0 otherwise	0.002 (0.000)
Peripheral vascular disease	Dummy variable = 1 if patient was diagnosed as peripheral vascular disease, 0 otherwise	0.001 (0.000)
Dementia	Dummy variable = 1 if patient was diagnosed as dementia, 0 otherwise	0.001 (0.000)
Chronic obstructive pulmonary disease	Dummy variable = 1 if patient was diagnosed as chronic obstructive pulmonary disease, 0 otherwise	0.014 (0.001)
Rheumatologic disease	Dummy variable = 1 if patient was diagnosed as rheumatologic disease, 0 otherwise	0.004 (0.001)
Peptic ulcer disease	Dummy variable = 1 if patient was diagnosed as peptic ulcer disease, 0 otherwise	0.073 (0.002)
Diabetes complicated	Dummy variable = 1 if patient was diagnosed as diabetes complicated, 0 otherwise	0.011 (0.001)
Diabetes uncomplicated	Dummy variable = 1 if patient was diagnosed as diabetes uncomplicated, 0 otherwise	0.294 (0.004)
Hemiplegia or paraplegia	Dummy variable = 1 if patient was diagnosed as hemiplegia or paraplegia, 0 otherwise	0.001 (0.000)
Renal disease	Dummy variable = 1 if patient was diagnosed as renal disease, 0 otherwise	0.089 (0.003)
Metastatic solid tumor	Dummy variable = 1 if patient was diagnosed as metastatic solid tumor, 0 otherwise	0.020 (0.001)
AIDS	Dummy variable = 1 if patient was diagnosed as AIDS, 0 otherwise	0.005 (0.001)
Cardiac arrhythmias	Dummy variable = 1 if patient was diagnosed as cardiac arrhythmias, 0 otherwise	0.034 (0.002)
Valvular disease	Dummy variable = 1 if patient was diagnosed as valvular disease, 0 otherwise	0.002 (0.000)
Hypothyroidism	Dummy variable = 1 if patient was diagnosed as hypothyroidism, 0 otherwise	0.007 (0.001)
Lymphoma	Dummy variable = 1 if patient was diagnosed as lymphoma, 0 otherwise	0.002 (0.000)
Blood loss anemia	Dummy variable = 1 if patient was diagnosed as blood loss anemia, 0 otherwise	0.141 (0.003)
Deficiency anemia	Dummy variable = 1 if patient was diagnosed as deficiency anemia, 0 otherwise	0.123 (0.003)
Depression	Dummy variable = 1 if patient was diagnosed as depression, 0 otherwise	0.005 (0.001)
Psychoses	Dummy variable = 1 if patient was diagnosed as psychoses, 0 otherwise	0.002 (0.000)
Renal failure	Dummy variable = 1 if patient was diagnosed as renal failure, 0 otherwise	0.022 (0.001)
solid tumor	Dummy variable = 1 if patient was diagnosed as solid tumor, 0 otherwise	0.217 (0.004)
Hypertension uncomplicated	Dummy variable = 1 if patient was diagnosed as hypertension uncomplicated, 0 otherwise	0.176 (0.004)
Hypertension complicated	Dummy variable = 1 if patient was diagnosed as hypertension complicated, 0 otherwise	0.101 (0.003)
Alcohol abuse	Dummy variable = 1 if patient was diagnosed as alcohol abuse, 0 otherwise	0.177 (0.004)

SE: standard error; RR: reimbursement ratio, RMT: ratio of medication cost to total hospital cost; LOS: length of stay at hospital; MELDNa: model for end-stage liver disease and sodium; AIDS: acquired immune deficiency syndrome; TACE: transcatheter arterial chemoembolization.

As shown in Table 3, after adjusting for liver disease severities, comorbidities and major procedures, the RR was found to be significantly correlated with LOS (1.051, p < 0.0001): patients with higher RR (i.e., more generous insurance coverage) were found to stay longer in hospital. Specifically, every 10% increase of RR was related to a 3% increase of LOS. We analyzed the effect of RR on the use of TACE among patients with PLC and/or cirrhosis including two outcomes: use or not and number of TACE. As presented in Table 4, our results showed that after adjusting for demographics and case mix, patients with a higher RR were significantly more likely to undergo TACE than patients with a lower RR (2.330, p < 0.0001), and also tended to have higher number of TACE (1.511, p = 0.025). This means that every 10% increase of RR is associated with a 0.15 increase in the number of TACE and a 30% increase of the odds of undergoing TACE. Our results show that in-hospital utilization for liver disease patients, with both demographics and case mix adjusted for, is consistently and significantly higher for patients with more generous insurance coverage.

**Table 3:** Effect of RR (coefficient and standard error) on hospital utilization.

Parameter	LOS	TACE use or not^	Number of TACE^
	N = 10,102	N = 4,736	N = 434
Intercept	1.051*** (0.019)	-1.604*** (0.482)	-0.534 (1.131)
RR	0.131*** (0.017)	2.330*** (0.519)	1.511** (0.676)
Age	0.004*** (0.000)	0.010** (0.005)	0.009 (0.008
Sex	-0.177*** (0.007)	-0.686*** (0.134)	-0.321 (0.198)
Admission status	-0.126*** (0.008)	-1.477*** (0.319)	-0.230 (0.502)
MELDNa score	-0.005*** (0.001)	-0.290*** (0.022)	-0.100*** (0.035)
Primary liver cancer	0.014* (0.008)	-	-
Cirrhosis	0.104*** (0.007)	-	-
Hepatitis B	-0.094*** (0.008)	0.474*** (0.146)	0.373 (0.250
Hepatitis C	-0.068*** (0.017)	-0.001 (0.288)	0.114 (0.443)
Alcoholic hepatitis	-0.031 (0.019)	-1.706*** (0.464)	0.219 (0.719)
Combined hepatitis (B, C or alcoholic)	0.013 (0.015)	-0.191 (0.280)	1.399*** (0.444)
Chronic obstructive pulmonary disease	-0.003 (0.023)	0.049 (0.360)	-0.466 (0.517)
Rheumatologic disease	0.004 (0.042)	0.467* (0.702)	-1.215 (1.053)
Peptic ulcer disease	0.081*** (0.011)	-0.412** (0.170)	-0.101 (0.261)
Diabetes complicated	-0.038 (0.028)	-0.881 (0.552)	-0.078 (0.878)
Diabetes uncomplicated	0.032*** (0.007)	0.183 (0.114)	0.424** (0.166)
Renal disease	0.040*** (0.012)	0.175 (0.196)	0.134 (0.305)
Metastatic solid tumor	-0.005 (0.020)	-0.553 (0.451)	-0.376 (0.700)
Cardiac arrhythmias	0.001 (0.015)	-0.283 (0.267)	-0.028 (0.404)
Hypothyroidism	0.073** (0.032)	-0.356 (0.630)	2.546*** (0.958)
Blood loss anemia	0.040** (0.019)	-0.091 (0.364)	0.339 (0.601)
Deficiency anemia	-0.015 (0.020)	-0.617 (0.435)	-0.802 (0.715)
Depression	0.024 (0.040)	-0.361 (0.754)	0.152 (1.274)
Renal failure	0.005 (0.021)	0.886*** (0.332)	-0.163 (0.477)
Solid tumor	-0.003 (0.008)	1.002** (0.404)	-0.204 (0.536)
Hypertension uncomplicated	0.018* (0.011)	0.060 (0.169)	0.177 (0.245)
Hypertension complicated	-0.012 (0.013)	0.021 (0.205)	0.136 (0.289)
Alcohol abuse	-0.061*** (0.015)	0.201* (0.299)	-1.068** (0.453)
Hepatectomy	0.120*** (0.031)	-1.351*** (0.474)	-0.980 (0.736)
TACE	0.097*** (0.015)	-	-
		c-statistic = 0.809	

SE: standard error; RR: reimbursement ratio, RMT: ratio of medication cost to total hospital cost; LOS: length of stay at hospital; MELDNa: model for end-stage liver disease and sodium; AIDS: acquired immune deficiency syndrome; TACE: transcatheter arterial.

**^** TACE is only for primary liver cancer and cirrhosis patients, so the primary liver cancer and cirrhosis are excluded when modeling the TACE use and the number of TACE

*** indicates that coefficient is significant at 1% level

** indicates that coefficient is significant at 5% level; and

* indicates that coefficient is significant at 10% level.

**Table 4:** Effect of RR (coefficient and standard error) on hospital cost.

Parameter	Total cost	Medication cost	MTratio
	N = 10858	N = 10858	N = 10858
Intercept	3.655*** (0.019)	2.815*** (0.044)	-0.773*** (0.025)
RR	0.049*** (0.017)	0.566*** (0.038)	0.439*** (0.022)
Age	0.004*** (0.001)	0.003*** (0.001)	-0.001** (0.000)
Sex	-0.096*** (0.007)	-0.099*** (0.016)	-0.016 (0.009)
Admission status	0.020** (0.009)	0.065*** (0.02)	0.036*** (0.011)
Primary liver cancer	0.224*** (0.009)	0.237*** (0.077)	0.030** (0.012)
Cirrhosis	0.159*** (0.008)	0.055 (0.037)	0.073*** (0.010)
Hepatitis B	-0.008 (0.009)	0.006 (0.02)	0.004 (0.011)
Hepatitis C	-0.030* (0.018)	-0.036 (0.041)	-0.014 (0.023)
Alcoholic hepatitis	0.001 (0.021)	0.011 (0.046)	-0.004 (0.026)
Combined hepatitis (B, C or alcoholic)	-0.006 (0.016)	-0.011 (0.035)	-0.015 (0.020)
Hepatectomy	0.249*** (0.034)	0.240*** (0.017)	-0.016 (0.044)
MELDNa score	-0.001* (0.001)	1.030*** (0.307)	-0.001 (0.001)
Chronic obstructive pulmonary disease	0.024 (0.025)	0.06 (0.056)	0.031 (0.032)
Rheumatologic disease	0.029 (0.046)	-0.084 (0.104)	-0.064 (0.059)
Peptic ulcer disease	0.087*** (0.012)	0.121*** (0.027)	0.034** (0.015)
Diabetes complicated	-0.062** (0.029)	-0.125* (0.066)	-0.056 (0.038)
Diabetes uncomplicated	0.084*** (0.007)	0.128*** (0.016)	0.036*** (0.009)
Renal disease	0.070*** (0.012)	0.061** (0.028)	-0.010 (0.016)
Metastatic solid tumor	0.02 (0.021)	0.083* (0.048)	0.053* (0.027)
Cardiac arrhythmias	-0.001 (0.017)	0.004 (0.037)	0.000 (0.021)
Hypothyroidism	0.036 (0.035)	0.068 (0.078)	0.030 (0.045)
Blood loss anemia	0.206*** (0.02)	0.265*** (0.046)	0.056** (0.026)
Deficiency anemia	-0.035 (0.022)	0.002 (0.049)	0.026 (0.028)
Depression	0.025 (0.041)	0.052 (0.094)	0.019 (0.053)
Renal failure	0.033 (0.023)	-0.015 (0.051)	-0.046 (0.029)
Solid tumor	0.000 (0.009)	-0.009 (0.02)	-0.009 (0.011)
Hypertension uncomplicated	0.025** (0.012)	0.042 (0.026)	0.009 (0.015)
Hypertension complicated	-0.027* (0.014)	-0.02 (0.032)	0.011 (0.018)
Alcohol abuse	0.029* (0.016)	0.082** (0.037)	0.050** (0.021)
TACE	0.134*** (0.016)	-0.003* (0.001)	-0.080*** (0.021)
LOS	0.010*** (0.000)	0.014*** (0.000)	0.004*** (0.000)

SE: standard error; RR: reimbursement ratio, RMT: ratio of medication cost to total hospital cost; LOS: length of stay at hospital; MELDNa: model for end-stage liver disease and sodium; AIDS: acquired immune deficiency syndrome; TACE: transcatheter arterial.

*** indicates that coefficient is significant at 1% level

** indicates that coefficient is significant at 5% level; and

* indicates that coefficient is significant at 10% level.

After adjusting for patient characteristics and case mix, RR is also significantly associated with total inpatient cost: patients with higher RR had a higher total hospital cost versus patients with a lower RR (0.049, p<0.0001) (Table 4). In addition, patients with a higher RR were found to incur significantly higher medication costs (0.566, p<0.0001). For patients with a higher RR, percentage of medication cost in their total inpatient cost (RMT) was also significantly higher (0.439, p<0.0001) after adjusting for other factors. In other words, when RR increases 0.1 (e.g., from 0.1 to 0.2), the total hospital cost, medication cost, and percentage of medication cost of total cost increases 1.0%, 10.0%, and 10.0%, respectively.

After adjusting for the disease severity, comorbidities and major procedures (such as hepatectomy and TACE), RR was not found to be significantly associated with patient outcome, measured by in-hospital mortality (p = 0.08, OR 1.87, 95% CI: 0.92–3.81) (See Table 5). Patient cost sharing did not seem to have led to significant differences in outcome among patients with different insurance coverage, as measured by in-hospital mortality. This result indicates that financial incentives may not impact the life-threatening outcomes (such as death) given that mortality is mainly related to clinical factors and health providers and patients reactions to financial incentives do not aim to and are not able to change such a disease outcome.

**Table 5:** Effect of RR (coefficient and standard error) on in-hospital mortality.

Parameter	Estimate (SE)	Odds Ratio (95% CI)
Intercept	-8.964*** (0.387)	-
RR	0.627* (0.362)	1.872 (0.92–3.808)
Age	0.045*** (0.004)	1.046 (1.038–1.055)
Male	-0.476*** (0.121)	0.621 (0.49–0.788)
Admission status	0.33*** (0.126)	1.391 (1.087–1.779)
Hepatitis B	-0.087 (0.130)	0.917 (0.711–1.183)
Hepatitis C	-0.218 (0.286)	0.804 (0.459–1.409)
Alcoholic hepatitis	-0.858** (0.352)	0.424 (0.213–0.846)
Combined hepatitis (B,C or alcoholic)	-1.057*** (0.296)	0.348 (0.195–0.621)
MELDNa score	0.184*** (0.010)	1.202 (1.179–1.226)
Cirrhosis	0.254** (0.111)	1.29 (1.038–1.603)
Primary liver cancer	1.422*** (0.111)	4.147 (3.334–5.159)
Chronic obstructive pulmonary disease	-0.219 (0.306)	0.803 (0.441–1.464)
Rheumatologic disease	-0.919 (0.684)	0.399 (0.104–1.524)
Peptic ulcer disease	-0.148 (0.159)	0.862 (0.631–1.178)
Diabetes uncomplicated	-0.932** (0.407)	0.394 (0.177–0.875)
Diabetes complicated	0.398*** (0.104)	1.489 (1.215–1.824)
Renal disease	0.657*** (0.132)	1.928 (1.488–2.499)
Metastatic solid tumor	0.211 (0.316)	1.234 (0.665–2.292)
Cardiac arrhythmias	0.247 (0.19)	1.281 (0.882–1.858)
Hypothyroidism	0.857** (0.410)	2.357 (1.055–5.267)
Blood loss anemia	1.266*** (0.201)	3.546 (2.392–5.257)
Deficiency anemia	-0.795*** (0.227)	0.452 (0.289–0.705)
Depression	-0.182 (0.580)	0.833 (0.268–2.594)
Renal failure	0.346* (0.201)	1.414 (0.954–2.097)
Solid tumor	0.376 (0.443)	1.456 (0.612–3.468)
Hypertension uncomplicated	-0.041 (0.166)	0.959 (0.693–1.328)
Hypertension complicated	-0.374* (0.197)	0.688 (0.468–1.012)
Alcohol abuse	0.645** (0.309)	1.906 (1.041–3.491)
Hepatectomy	-1.451 (1.021)	0.234 (0.032–1.735)
TACE	-0.498** (0.238)	0.607 (0.381–0.969)
**Model diagnostics**	**N = 10858**	**c-statistic = 0.915**

SE: standard error; CI: confidence interval; RR: reimbursement ratio, RMT: ratio of medication cost to total hospital cost, LOS: length of stay at hospital, MELDNa: model for end-stage liver disease and sodium, AIDS: acquired immune deficiency syndrome, TACE: transcatheter arterial.

*** indicates that coefficient is significant at 1% level;

** indicates that coefficient is significant at 5% level; and

* indicates that coefficient is significant at 10% level.

## Discussion

Using rich EMR data with detailed clinical and financial information from China on inpatients with liver disease, our results showed that after controlling for patient characteristics and case mix, patients with more generous health insurance coverage were found to have significantly higher LOS, higher total hospital cost, higher medication cost, higher ratio of medication cost, higher likelihood of having TACE performed and a higher number of TACE performed. Addressing some of the limitations in former studies, our study contributes to the existing literature by providing clear empirical evidence for the potential impact of patient cost-sharing on health care utilization, cost and outcome.

The current health system in China provides strong financial incentives for physicians to make decisions based not only on patient health status (i.e., with or without insurance) but also the generosity of patient health insurance coverage. Our findings are consistent with the Rand Health Insurance Experiment (HIE) which demonstrated the effect of patient cost-sharing on the use of health care, except that the observed effect of patient cost-sharing on health care utilization and cost in our study may be explained by a joint effect of not only demand but also supply side moral hazard.

The unique health insurance system in China has allowed us to study the impact of patient cost-sharing in a setting where there are large variations in insurance coverage and limited selection bias in insurance plan choice. The validity of this study was also greatly enhanced by the use of actual reimbursement ratio and through employing a robust risk adjustment method which was validated in our previous study [36]. This risk adjustment method considered the important risk factors for health care utilization, cost and outcome including patient disease severity and comprehensive comorbidities.

Our study has a few limitations. First, generalizability of the study result may be a concern, given that we only used EMR data from one hospital. Moreover, in this retrospective observational study the variation of RR may not be entirely random and might contribute to potential selection bias. However, YouAn hospital is a well-known liver disease-specializing hospital treating patients from all regions of China. Such wide geographic coverage would mitigate any potential selection bias. Second, the EMR data contained large amounts of missing values in demographic and socioeconomic data such as income level, education and employment status. As a result, these potential confounding factors were not adjusted in our analyses. Third, using only one hospital’s data may not capture all the hospitalizations for one patient if the patient was admitted to other hospitals during the study period. This may affect the analysis on TACE use if the patient had undergone TACE in another hospital. However, this situation may not count for a large proportion among the sample, given that YouAn hospital is one of the leading liver disease centers with very high volume of TACE treatments and attracts a lot of liver diseases patients who are specifically seeking this treatment. Finally, our results indicated that patient cost-sharing did not lead to significant differences in patient outcomes. However, our outcome measure only included in-hospital mortality, which did not capture long term disease outcomes.

## Conclusion

In summary, our study implied that patient cost-sharing affects health care use and cost. This reflects how patients and physicians respond to financial incentives in the current healthcare system in China, and could be a joint effect of both demand and supply side moral hazard. In order to contain cost and improve efficiency in the system, reforming provider payment and insurance coverage is urgently needed.
