# Correction: The effects of patient cost sharing on inpatient utilization, cost, and outcome

**DOI:** 10.1371/journal.pone.0196039

**Published:** 2018-04-13

**Authors:** Yuan Xu, Ning Li, Mingshan Lu, Elijah Dixon, Robert P. Myers, Rachel J. Jolley, Hude Quan

The sixth author’s name is spelled incorrectly. The correct name is: Rachel Joy Jolley. The correct citation is: Xu Y, Li N, Lu M, Dixon E, Myers RP, Jolley RJ, et al. (2017) The effects of patient cost sharing on inpatient utilization, cost, and outcome. PLoS ONE 12(10): e0187096. https://doi.org/10.1371/journal.pone.0187096
